# Seroprevalence of influenza D virus in selected sample groups of Irish cattle, sheep and pigs

**DOI:** 10.1186/s13620-019-0150-8

**Published:** 2019-10-29

**Authors:** Tom O’Donovan, Leah Donohoe, Mariette F. Ducatez, Gilles Meyer, Eoin Ryan

**Affiliations:** 1 Central Veterinary Research Laboratory, Celbridge, Co. Kildare Ireland; 2 INRA UMR 1225 IHAP-ENVT, 31076 Toulouse, France; 3 Ruminant Animal Health Division, Department of Agriculture, Food and the Marine, Celbridge, Ireland

**Keywords:** Influenza, Cattle, Virus, Respiratory, Surveillance

## Abstract

Influenza D virus (IDV) is a new member of the Orthomyxoviridae family. It was first reported in swine in 2011 and isolated from bovine samples received for routine respiratory disease diagnosis in Ireland during 2014–2016. The goal of this study was to determine the seroprevalence in selected populations of IDV in cattle, pigs and sheep. Results showed a high prevalence of IDV in cattle sampled at slaughter (94.6%) or for diagnostic reasons (64.9%), whereas prevelance in samples taken for diagnostic reasons from sheep (4.5%) and pigs (5.8%) was much lower. This study suggests that IDV is widespread in Irish cattle.

## Introduction

Influenza viruses are enveloped RNA viruses that are members of the *Orthomyxoviridae* family [1]. The natural reservoir of influenza A virus (IAV) is waterfowl and as well as causing outbreaks in poultry, it is responsible for both seasonal and pandemic influenza in humans [2]. The main reservoir of influenza B virus is humans and it causes seasonal influenza [3]. Influenza C virus causes milder disease, primarily lower-respiratory-tract infections in children [4]. In 2011, a novel influenza virus was isolated from swine in America that was designated influenza D virus (IDV). It was shown to share approximately 50% sequence similarity with influenza C virus [5]. Although this was first detected in swine, surveillance data suggest the natural reservoir for this virus is cattle [6]. IDV has subsequently been detected in several European countries including Italy and France [7, 8]. IDV was detected in Irish cattle submitted for routine diagnosis during 2014–2016 [9] and on this basis a seroprevalence study was carried out to determine the prevalence of IDV in Irish cattle. A smaller number of swine and ovine samples were also tested for the presence of IDV antibodies.

## Materials and methods

This study used 1219 bovine serum samples taken at slaughter from healthy beef cattle aged 30–36 months which had passed ante-mortem veterinary inspection. These samples were taken in January 2017 from a range of slaughter plants across Ireland to ensure a representative geographical spread. In addition, 1183 serum samples from cattle were included which had been taken during 2016 and early 2017 for diagnostic purposes to screen for antibodies to bovine respiratory disease (BRD) pathogens were used. A smaller number of swine and ovine sera, 377 and 288 respectively, were also included in the study. The swine and ovine sera had been submitted for routine general diagnostic testing. The number of samples selected was based on availability rather than design prevalence; the samples taken at slaughter were originally selected for surveillance for another disease, while samples submitted for diagnostic purposes from cattle, sheep and pigs were used as convenience samples rather than random samples.

Each sample was tested for antibodies to influenza D virus. Haemagglutination Inhibition (HAI) assay was performed as described in standard protocols [10]. Briefly, sera were inactivated with receptor-destroying enzyme (RDE), 50uL of sera to 200uL of RDE, and incubated overnight at 37 °C. 200uL of 1.5% sodium citrate was added to each sample and heat-inactivated at 56 °C for 30 min. Finally, sera were treated with 50uL of 50% Turkey red blood cells to give a final dilution of 1 in 10. HAI assay was performed using 0.75% Turkey red bloods cells in V-well plates. The HAI assay was then conducted using the stock virus D/Bovine/France/5920/2014. A homologous positive control serum was also included in the assay. A 1 in 40 dilution of the stock virus was required to produce a working dilution of 4HAU. Samples with titres of ≥40 were considered positive as per previous studies [6]. Serological cross reactivity against influenza C virus was not considered as it has been previously demonstrated that no cross reactivity between these two viruses is present [5].

## Results

Of the 1219 samples collected randomly from healthy beef cattle at routine slaughter, 1153 were positive for antibodies to IDV, resulting in a seroprevalence of 94.6% (95% confidence interval 95.87, 93.33%). A lower seroprevalence of 64.9% was observed in the samples taken from cattle for diagnostic testing for BRD; 768 positive samples from a total of 1183 tested. A breakdown of the titres observed in positive bovine samples is as follows; a 1/40 titre in 7% of samples, a 1/80 titre in 15% of samples, a 1/160 titre in 23% of samples, a 1/320 titre in 19% of samples, a 1/640 titre in 11% of samples, a 1/1280 in 3% of samples, 1/2560 in 1% of samples and 1/5120 in 0.2% of samples. Finally, 0.2% of samples had a titre ≥1/10240. Swine and ovine serum had much lower prevalence; 5.8% for swine and 4.5% for ovine samples. Confidence intervals were not calculated for the diagnostic samples since they were non-random submissions.

## Discussion

The results reported in this study are important as they establish that IDV infection in the Irish cattle population is far more widespread than previously thought, and that cattle are a more important host for this virus, rather than pigs or sheep. It is notable that broadly similar findings were reported from Luxembourg, where 80.2% seroprevalence was reported in cattle [11].

It has previously been reported that IDV may have a role to play in the BRD complex [12]. Therefore, it might be expected that a higher prevelance of IDV antibodies would be observed in cattle samples taken for diagnostic testing for BRD than in samples taken from healthy cattle at slaughter. However, the data show the reverse to be the case. This finding may be the result of an age difference in the two groups. Although one of the limitations of the study is that animal-level age data was not available for diagnostic submissions, the beef cattle which were sampled at slaughter were all aged 30 to 36 months; in contrast, samples submitted for routine BRD diagnostic testing are most frequently younger animals, including calves and weanlings. Age may therefore be acting as a confounder in comparing the two groups; as cattle get older, they may have more opportunities to become exposed and infected with IDV. However, the multifactorial aetiology and epidemiology of BRD means that it is important to take a nuanced approach to interpreting these results; it may be that IDV plays a role as a co-factor or potentiating agent in BRD in Irish cattle, and that it also spreads through the clinically normal population in such a way as to result in the remarkably high prevalence reported here. Further research is necessary to understand the epidemiology and any clinical impact of IDV in cattle.

The prevalence rates for pigs and sheep were much lower and the pig data were in line with those reported in other European countries. Italy recorded a prevalence in pigs of 11.7% in 2015 [13], Luxembourg of 5.9% in 2014–2015 [11].

The zoonotic potential of IDV is not yet clear but as it is closely related to ICV, the possibility is worth considering. Given the high prevalence observed in Irish cattle, further investigation in people with increased exposure to cattle may be warranted.

This study has demonstrated that infection with IDV is widespread in two populations of Irish cattle studied (beef cattle aged 30–36 months and cattle sampled for diagnostic reasons), but has a relatively low prevalence in populations of Irish sheep and pigs which have been sampled for diagnostic reasons. Further research is needed to address the role IDV may play in bovine respiratory disease and to explore the potential for any zoonotic risk.

## Data Availability

The datasets used and/or analysed during the current study are available from the corresponding author on reasonable request.

## Abbreviations

BRD: Bovine respiratory disease
HAI: Haemagglutination Inhibition
IAV: Influenza A virus
IBV: Influenza B virus
ICV: Influenza C virus
IDV: Influenza D virus
RDE: Receptor-destroying enzyme
